# Reply: Inflammatory breast carcinoma as a model of accelerated self-metastatic expansion by intra-vascular growth

**DOI:** 10.1038/sj.bjc.6605252

**Published:** 2009-08-25

**Authors:** H Enderling, L Hlatky, P Hahnfeldt

**Affiliations:** 1 Center of Cancer Systems Biology, Caritas St Elizabeth's Medical Center, Tufts University School of Medicine, 736 Cambridge Street, Boston, MA 02135, USA

**Sir,**

We are encouraged by the clinico-pathological evidence provided by Vermeulen *et al* (2009) in support of the self-metastasis concept, and in particular, that the self-metastatic dynamic is a logical mechanism for understanding a characteristic invasive fingering morphology driven by migratory cell types in inflammatory breast carcinoma (IBC). In their argument, Vermeulen *et al* re-emphasise the importance of cancer stem cells in the *in silico* model to seed new separate clusters (Enderling *et al*, 2009), and that a stem-cell-like phenotype is expressed in lymphovascular emboli *in vivo* as well as in IBC patients. The clinical features of IBC – fast local growth and efficient metastasis (with almost all patients having lymph node metastases and about one-third having distant metastases) – motivate a straightforward extension of the self-metastasis model to also explain the formation of distant metastases. Tumours that grow in a self-metastatic manner have a large distribution of stem cells in self-seeded clusters at the tumour periphery, and it is these cancer stem cells that would potentially intravasate into nearby vessels to drive intra-vascular growth and seed distant metastases. In a preliminary test of this idea, we found that self-metastatic tumours have a preferred tendency to shed cancer stem cells well into extra-tumoral tissue, where they can either form local self-metastases or enter the blood stream to potentially form distant metastases. Tumours that do not exhibit the self-metastatic phenotype, by contrast, prove to be slow growing and unable to seed stem cells either to areas adjacent to the primary site or to distant sites. In closing, we appreciate the contribution of Vermeulen *et al* in providing very important clinical and pathological correlations that further support the self-metastasis concept, and by extension, a more unified basis for understanding local and distant tumour dissemination.
